# Sodium-Vanadium Bronze Na_9_V_14_O_35_: An Electrode Material for Na-Ion Batteries

**DOI:** 10.3390/molecules27010086

**Published:** 2021-12-24

**Authors:** Maria A. Kirsanova, Alexey S. Akmaev, Mikhail V. Gorbunov, Daria Mikhailova, Artem M. Abakumov

**Affiliations:** 1 Center for Energy Science and Technology, Skolkovo Institute of Science and Technology, Nobel Str. 3, 121205 Moscow, Russia; Aleksei.Akmaev@skoltech.ru (A.S.A.); a.abakumov@skoltech.ru (A.M.A.); 2 Leibniz Institute for Solid State and Materials Research Dresden, Institute for Complex Materials, Helmholtzstraße 20, 1069 Dresden, Germany; m.gorbunov@ifw-dresden.de (M.V.G.); d.mikhailova@ifw-dresden.de (D.M.)

**Keywords:** Na-ion batteries, sodium-vanadium bronzes, electrochemical cycling

## Abstract

Na_9_V_14_O_35_ (η-Na_x_V_2_O_5_) has been synthesized via solid-state reaction in an evacuated sealed silica ampoule and tested as electroactive material for Na-ion batteries. According to powder X-ray diffraction, electron diffraction and atomic resolution scanning transmission electron microscopy, Na_9_V_14_O_35_ adopts a monoclinic structure consisting of layers of corner- and edge-sharing VO_5_ tetragonal pyramids and VO_4_ tetrahedra with Na cations positioned between the layers, and can be considered as sodium vanadium(IV,V) oxovanadate Na_9_V_10_^4.1+^O_19_(V^5+^O_4_)_4_. Behavior of Na_9_V_14_O_35_ as a positive and negative electrode in Na half-cells was investigated by galvanostatic cycling against metallic Na, synchrotron powder X-ray diffraction and electron energy loss spectroscopy. Being charged to 4.6 V vs. Na^+^/Na, almost 3 Na can be extracted per Na_9_V_14_O_35_ formula, resulting in electrochemical capacity of ~60 mAh g^−1^. Upon discharge below 1 V, Na_9_V_14_O_35_ uptakes sodium up to Na:V = 1:1 ratio that is accompanied by drastic elongation of the separation between the layers of the VO_4_ tetrahedra and VO_5_ tetragonal pyramids and volume increase of about 31%. Below 0.25 V, the ordered layered Na_9_V_14_O_35_ structure transforms into a rock-salt type disordered structure and ultimately into amorphous products of a conversion reaction at 0.1 V. The discharge capacity of 490 mAh g^−1^ delivered at first cycle due to the conversion reaction fades with the number of charge-discharge cycles.

## 1. Introduction

A commercial application of rechargeable sodium-ion batteries (SIBs) would bring substantial alleviation and expansion of the existing energy storage market, which is mainly based on the Li-ion battery (LIB) technology. In terms of material abundance, SIBs appear to be the cheaper alternative to LIBs that enables their usage in high-scale energy storage, for instance, in smart-grid applications. The positive electrode (cathode) materials, especially the layered transition metal oxides, are an intensively studied topic in the field of SIBs, mostly because of the fact that the overall battery energy and power density are primarily limited by the cathode material. Since the ionic volume of sodium is about 70% larger than that of lithium, the structural chemistry of the Na-ion (de)intercalation systems is more complicated compared to the Li-based ones as the size difference of Na^+^ and transition metal cations M^2+^ or M^3+^ is quite large, demanding higher flexibility of the hosting frameworks. State-of-the art cathode materials for SIBs belong to two large groups: layered and 3-dimensional 3d and 4d metal oxides and polyanion structures (phosphates, sulphates, etc.) [1,2].

**Figure 1:**
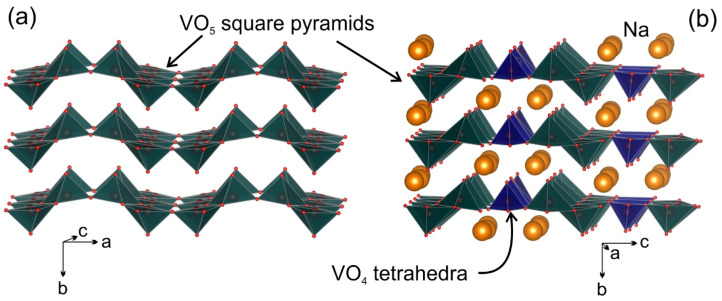
Polyhedral presentation of the crystal structure of V_2_O_5_ (**a**) and Na_9_V_14_O_35_ (**b**).

Vanadium pentoxide V_2_O_5_, belonging to the family of 2D oxides, was studied as an insertion structure for both sodium and lithium ions [3]. A family of sodium-vanadium bronzes with the general formula Na_x_V_2_O_5_ (0 < x ≤ 2) with mixed valence of the vanadium ions between V^4+^ and V^5+^, unlike the bronzes of other transition metals, comprises a wide variety of structures termed α-, β-, γ-, δ-, τ-, α’-, η-, κ and χ [4,5,6,7,8,9,10]. The general structure motive for the Na_x_V_2_O_5_ bronzes is adopted from the layered structure of V_2_O_5_, which is built of edge- and vertex-sharing VO_5_ square pyramids (Figure 1a), though the crystal structures of the bronzes are exceptionally flexible as exemplified with the layered (α-phase) and tunnel (β-phase) materials [11,12]. Among sodium-based vanadium bronzes, monoclinic β-Na_0.33_V_2_O_5_ has gained much attention because of tunnel structure, adopting three different Na intercalation sites and ensuring good structural reversibility even upon deep charge/discharge. Numerous attempts in preparation of nanostructured β-Na_0.33_V_2_O_5_ resulted in impressive discharge capacity above 300 mAh g^−1^ in the Li-ion cells [13,14,15,16]. The cycling performance is highly dependent on the particle’s morphology, potential window and current density, and the best compromise between these electrochemical characteristics seems to be reached for the micro-rod β-Na_0.33_V_2_O_5_ material showing 297 mAh g^−1^ discharge capacity at low current density (1.5–4.0 V vs. Li^+^/Li), which is retained with a high efficiency after at least 50 cycles [13].

The α-V_2_O_5_ matrix could adopt various phase transformations during Li^+^ (de)intercalation. For example, the reduction of V_2_O_5_ in an anodic range below 1.9 V [17] results in formation of the Li_3_V_2_O_5_ phase with a disordered rock-salt structure, which can be reversibly cycled between 0.01 V and 2 V with a specific discharge capacity of 266 mAh g^−1^ (current density 0.1 A g^−1^) [18]. Impressive cycling performance of Li_3_V_2_O_5_ is preserved even at higher cycling rates, demonstrating the discharge capacity of 200 mAh g^−1^ after 1000 cycles at 1 A g^−1^.

Information on electrochemical behavior of vanadium bronzes or V_2_O_5_ in Na cells is still limited. γ-Na_x_V_2_O_5_ (x = 0.96; 0.97) synthesized by electrochemical reduction of γ’-V_2_O_5_ exhibits an orthorhombic layered structure (S.G. *Pnma*) related to the parent structure of V_2_O_5_, and shows specific capacities between 80–125 mAh g^−1^ in the one-step sodium-extraction-insertion process at 3.3–3.4 V vs. Na^+^/Na [7,9]. Muller-Bouvet et al. studied the electrochemical behavior of α’-NaV_2_O_5_, which was electrochemically formed during discharge of V_2_O_5_ in Na cell in the 3.0–1.6 V potential range. The α’-NaV_2_O_5_ bronze with an orthorhombic structure, which delivers a specific capacity of 120 mAh g^−1^ at 0.1 mA cm^−2^ current density, is also suitable for reversible sodium intercalation [19]. High discharge capacity of 250 mAh g^−1^ retained with 88% efficiency after 320 cycles at 20 mA g^−1^ (3.8–1.5 V vs. Na^+^/Na) was reported for so-called “bilayered” V_2_O_5_ with short-range ordering in the crystal structure, though no structural data were provided for the Na-containing phases formed during the reversible (de)intercalation process [20]. Nanostructured Na_0.33_V_2_O_5_ tested as an electrode material within the potential window of 1.5–4.0 V vs. Na^+^/Na demonstrated capacity of 130 mAh g^−1^ at the first discharge, and it showed gradual decay up to 90 mAh g^−1^ after 50 cycles at a 50 mA g^−1^ current density [21].

Inspired by high specific capacities and long cycling performance of vanadium bronzes, on the one hand, and the lack of a comprehensive study of vanadium bronzes in Na cells, on the other hand, we tailored this study to investigate the η-Na_x_V_2_O_5_ (x ~ 1.29) bronze as the host structure for (de)intercalation of Na cations. η-Na_x_V_2_O_5_ or Na_9_V_14_O_35_ crystallizes in the monoclinic lattice with the space group *P*2/*c* [22,23] and adopts a crystal structure built of (010) layers formed by VO_5_ (V^4+^) square pyramids and VO_4_ (V^5+^) tetrahedra, with the sodium atoms embedded between the layers (Figure 1b). Similarly to the mixed-valence sulfates, phosphates and other polyanion structures, Na_9_V_14_O_35_ can be considered as sodium vanadium(IV,V) oxovanadate Na_9_V_10_^4.1+^O_19_(V^5+^O_4_)_4_. Theoretical capacity of η-Na_x_V_2_O_5_ in case of extraction of all sodium atoms can be estimated as ~163 mAh g^−1^ that, being complemented with possible electrochemical activity in the anodic area, makes this material of potential interest as a new intercalation system for Na ions. In this paper, we report on synthesis, multidisciplinary study of Na_9_V_14_O_35_ as both positive and negative electrode materials in Na half-cells and evolution of the crystal structure upon gradual uptake of sodium atoms in the low-voltage range.

## 2. Results

### 2.1. Compositional and Crystallographic Characterization

**Figure 2:**
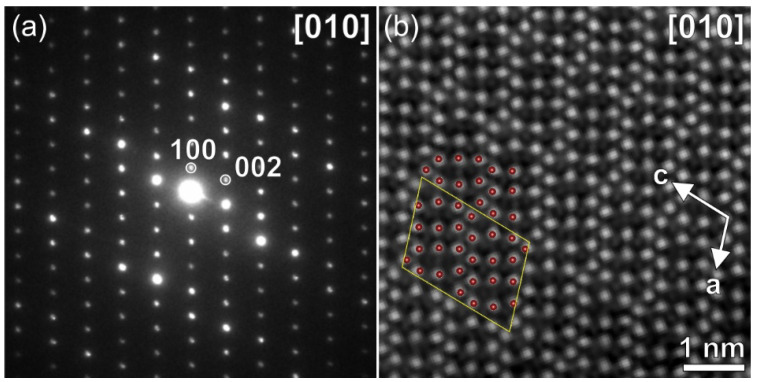
[010] SAED pattern (**a**) and [010] HAADF-STEM image (**b**) with superimposed projection of the crystal structure of pristine Na_9_V_14_O_35_. Ruby-colored spheres correspond to V atoms, O and Na atoms are not shown for clarity. The yellow parallelogram outlines the unit cell. White arrows show crystallographic axes of the monoclinic unit cell.

The PXRD pattern of Na_9_V_14_O_35_ was indexed with a monoclinic unit cell with parameters *a* = 15.1990(2) Å, *b* = 5.03271(4) Å, *c* = 20.7739(2) Å, β = 109.1635(6)°. The 00*l*, *l* = 2n reflection condition observed in the [010] SAED pattern (Figure 2a) unambiguously confirms the *P*2/*c* space group. Rietveld analysis (Figure S1, Table 1, Tables S1 and S2) was performed using a structure model reported by Isobe et al. [23]. The [010] HAADF-STEM image of Na_9_V_14_O_35_ demonstrates a well-ordered structure, where vanadium atomic columns appear as prominent bright dots, while the sodium columns are visible as faint dots, due to the difference in atomic numbers of V and Na (Figure 2b).

**Figure 3:**
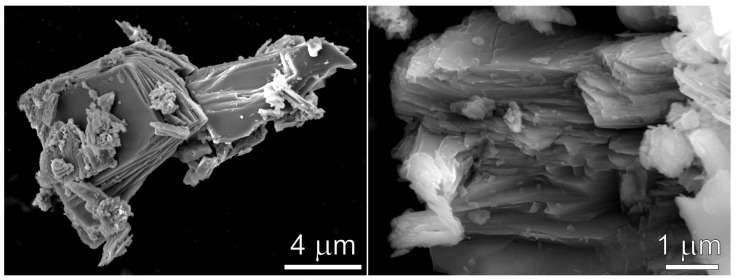
SEM images of polycrystalline Na_9_V_14_O_35_.

**Figure 4:**
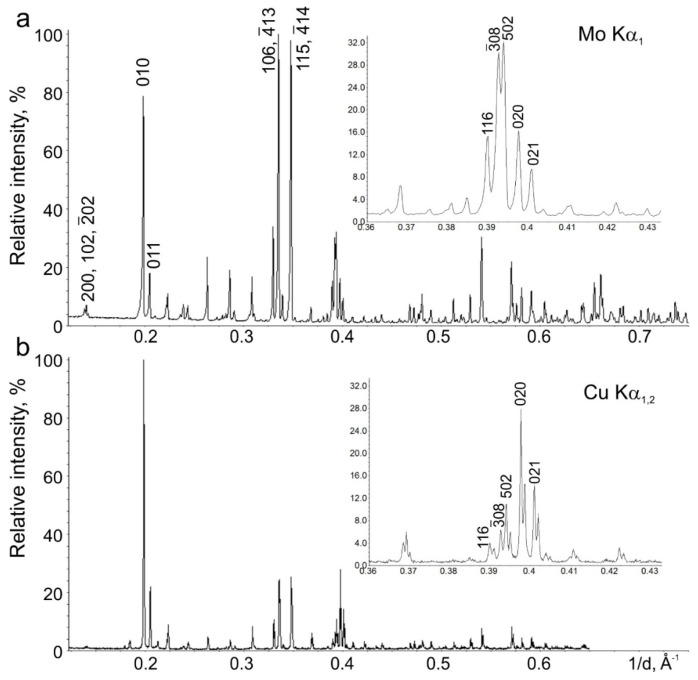
PXRD patterns of Na_9_V_14_O_35_ measured with diffractometers with a transmission geometry (**a**) and a reflection geometry (**b**) demonstrate strong preferred orientation of plate-like crystallites along the [010] direction.

The synthesized polycrystalline Na_9_V_14_O_35_ powder consists of thin platelets stacked into micron-sized agglomerates (Figure 3). The observed morphology corroborates with the strong preferred orientation effect visible as drastic difference in reflection intensities in the PXRD diffraction patterns collected with the diffractometers with the transmission and reflection (Bragg-Brentano) geometry (Figure 4) due to different angular relations between the preferred orientation axis **d**_t_ and reflection vector **H**_hkl_. The observed intensity variation indicates the preferred orientation of platelets perpendicular to the *b* axis. The EDX compositional maps of Na_9_V_14_O_35_ demonstrate homogeneous distribution of sodium and vanadium cations (Figure S2 of SI).

### 2.2. Electrochemical Characterization

As mentioned in Section 4, the active material was ball-milled to reduce the particle size. However, the discharge capacity in the first discharge cycle was only ~32 mAh g^−1^ for the 1.5–4.8 V potential window (Figure S3). The long plateau during the first charge above ~4.6 V is tentatively attributed to the electrolyte oxidation, thus further tests were limited by the 4.6 V threshold.

**Figure 5:**
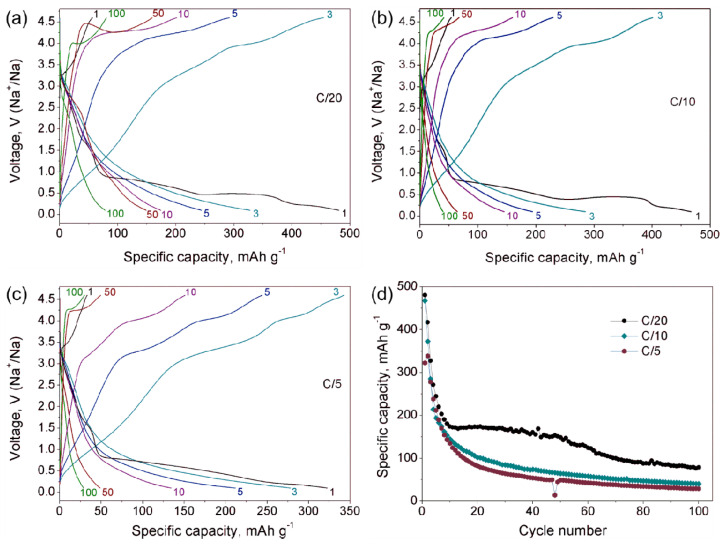
Galvanostatic charge-discharge curves of Na_9_V_14_O_35_ in Na cells at C/20 (**a**), C/10 (**b**) and C/5 (**c**) current rates. (**d**) Dependence of the specific discharge capacity on cycle number for different C-rates.

Galvanostatic cycling in the 0.1–4.6 V potential window was performed at C/20, C/10 and C/5 rates (Figure 5a–c). The shape of galvanostatic curves looks quite similar for the C/20 and C/10 rates and can be characterized by a low charge capacity in the first cycle (~50–60 mAh g^−1^) and high discharge capacity of 480 mAh g^−1^ with three well-pronounced plateaus in the first discharge curve. The charge capacity of 55 mAh g^−1^ corresponds to extraction of three sodium atoms per unit cell, what perfectly matches the results of quantitative EDX analysis, showing the Na:V = 6:14 atomic ratio for the material charged to 4.6 V (Table 2). The plateau located around 1.6 V is sloping, in contrast to two more flat plateaus at 0.8 and 0.5 V. The absence of any plateau in the second and all further discharge cycles accompanied by a fast fading of specific capacity (Figure 5d) indicates the possible conversion mechanism with fast structural degradation. At the same time, one can notice the growing charge capacity up to third cycle followed by its fading on the next cycles. This means that at first cycle, the extra sodium atoms, incorporated into the crystal structure upon discharge, can be reversibly extracted upon charge. Starting from the fourth cycle, the structural degradation prevails and hinders the (de)intercalation process, which is clearly represented by gradual fading of discharge capacity vs. cycle number (Figure 5d). The situation is quite similar for the material cycled at C/5 rate (Figure 5c), but the plateaus at 0.8 and 0.5 V are not so evident here, indicating kinetic limitations. To minimize the effect of the high cell potential on structural degradation, we performed galvanostatic cycling in the anodic area (Figure S4), but the resulting curves show the same tendency.

## 3. Discussion

**Figure 6:**
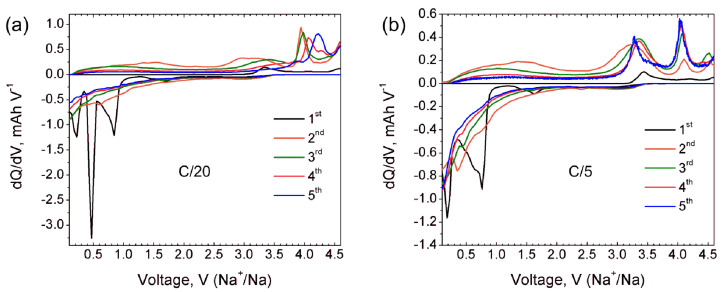
dQ/dV curves of Na_9_V_14_O_35_ in Na half-cell for the first five cycles at C/20 (**a**) and C/5 (**b**) current rates.

Additional information about the electrochemical processes involved during Na (de)intercalation is provided by dQ/dV curves at the C/20 rate (black curve in Figure 6a). The first cycle dQ/dV curve shows a broad cathodic peak at 3.3–3.4 V corresponding to the Na extraction, and an incomplete process of further Na extraction at 4.6 V. The first cathodic peak matches well with the (de)intercalation potential reported for γ-Na_x_V_2_O_5_ [7,9]. Anodic dQ/dV curve at the first discharge reveals Na insertion at potentials of 0.85 V, 0.48 V and 0.25 V. The origin of a small broad peak near 1.5 V is ambiguous and can be tentatively interpreted as a minor amount of embedded Na. dQ/dV curves for further cycles confirm the irreversible character of the Na insertion during the first discharge and no anodic peaks are observed anymore. At the same time, the cathodic curves for second and third cycles reveal a sharp peak at 3.9 V which becomes broader and shifts towards 4.0 V and 4.2 V at fourth and fifth cycles, respectively. These well-pronounced cathodic peaks indicate quantitative sodium extraction from the structure formed upon the first discharge.

**Figure 7:**
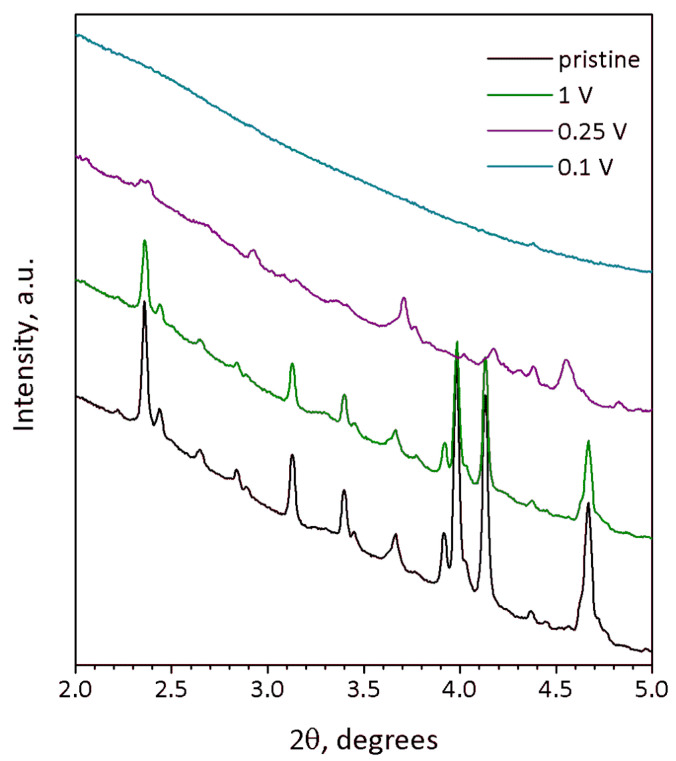
SXRD profiles of Na_9_V_14_O_35_ electrodes at different state of charge: pristine (black), discharged to 1 V (green), 0.25 V (violet) and 0.1 V vs. Na^+^/Na (turquoise). Wavelength λ = 0.20736 Å.

The degradation of the crystal structure supposed from galvanostatic curves is corroborated by ex situ SXRD (Figure 7) of Na_9_V_14_O_35_ at different states of discharge. The SXRD pattern of the material discharged to 1 V still looks identical to that of the pristine material and reveals close unit cell parameters *a* = 15.206(1) Å, *b* = 5.0325(8)(1) Å, *c* = 20.781(2) Å, β = 109.166(5)°, V = 1502.1(4) Å^3^ (Figure S5). The difference of unit cell volumes for the pristine and 1-V-discharged compounds is about 0.6 Å^3^ and falls into the range of two standard deviations, keeping in mind the unit cell volume of 1500 Å^3^ (Table 1). The absence of a significant change of the unit cell volume corresponds to a low discharge capacity of ~60 mAh g^−1^ registered at 1 V, since no significant amount of sodium has been intercalated into the structure at this potential. Moreover, [010] SAED pattern and HAADF-STEM image taken from the 1-V-discharged material (Figure S6) are obviously identical to those of pristine Na_9_V_14_O_35_. Both SXRD and TEM data correlate with the dQ/dV curve in Figure 6a, showing that no considerable Na insertion occurs above 1 V.

**Figure 8:**
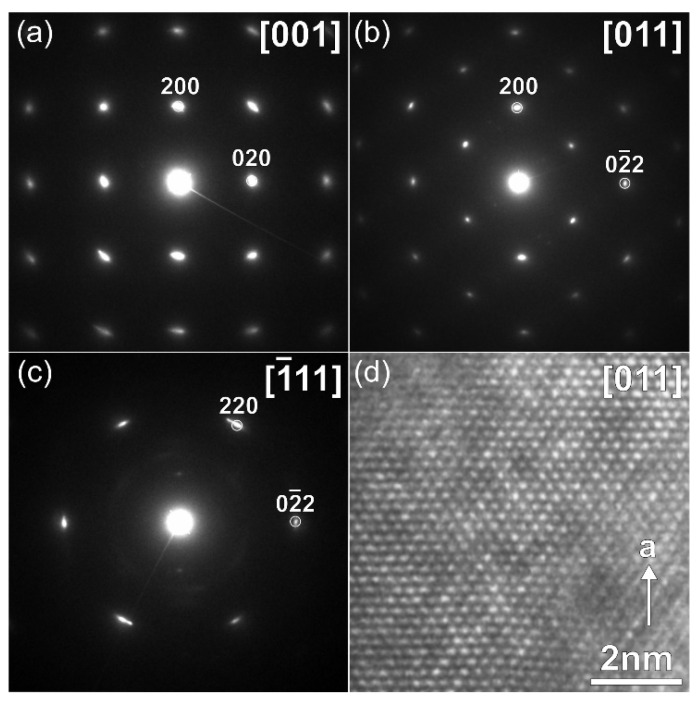
SAED patterns (**a**–**c**) indexed in the *F*-centered cubic lattice and [011] HAADF-STEM image (**d**), corresponding to DRS structure formed during discharge of Na_9_V_14_O_35_ to 0.25 V vs. Na^+^/Na.

The SXRD pattern of the 0.25-V-discharged material (Figure 7, violet curve) demonstrates suppressed but still visible reflections, the positions of which do not match those of pristine Na_9_V_14_O_35_. Although the quality of the SXRD pattern is insufficient for the Rietveld refinement, the reflections still can be indexed with the *P*2/*c* unit cell of Na_9_V_14_O_35_, but with a much larger *b*-parameter resulting in an about 31% increase of the unit cell volume: *a* = 15.347(3) Å, *b* = 6.301(1) Å, *c* = 21.367(3) Å, β = 107.253(9)°, V = 1973.3(8) Å^3^ (Figure S7). The expansion of the structure along the *a*- and *c*-axes is small (0.9% and 2.8%, respectively), but the expansion along the *b*-axis is huge and amounts to ~25.2%. This difference reflects the rigidity of the (010) layers formed by tightly interlinked VO_5_ square pyramids and VO_4_ tetrahedra, in which the expansion in the *a*-*c* plane occurs through elongation of the V-O bonds upon reduction of vanadium cations with increase in their ionic radius (r(V^2+^) = 0.79 Å, r(V^3+^) = 0.64 Å, r(V^4+^) = 0.58 Å, r(V^5+^) = 0.54 Å, CN = 6) [24]. Large increase in the interlayer separation is in line with the necessity to provide enough space to accommodate large amounts of Na, as the specific capacity at 0.25 V exceeds 400 mAh g^−1^. This must cause structure instability, and indeed, the crystals with another symmetry were found in the 0.25 V-discharged material, as one can see from the SAED patterns and high-resolution HAADF-STEM images, which are typical for a disordered rock-salt (DRS) structure with *F*-centered cubic lattice, a unit cell parameter *a* ~ 4.7 Å (Figure 8) and a Na:V ≈ 1:1 atomic ratio (Table 2). This observation is in agreement with the formation of the DRS structure, in which Na and V atoms randomly occupy the same crystallographic positions. The formation of the DRS structure was observed earlier in the related Li-ion system [18], in which it demonstrated a stable cycling at anodic potentials. However, the conversion process continues further in Na_9_V_14_O_35_ up to 0.1 V resulting in a complete amorphization as indicated by absence of any reflections in the corresponding SXRD pattern (Figure 7, turquoise curve). Regarding the Na and V distribution, the 0.1-V-discharged sample is strongly inhomogeneous. It still contains particles with the Na:V ≈ 1:1 atomic ratio (Table 2), but another phase, strongly enriched with Na up to Na:V ≈ 6.7:1 (Table 2, Figure S8), also appears in the sample, and is probably responsible for the high capacity of 490 mAh g^−1^. The multicomponent nature of active material at 0.1 V and progressing structural degradation upon further cycles cause the fast decrease of discharge capacity even at low current density (Figure 5d).

**Figure 9:**
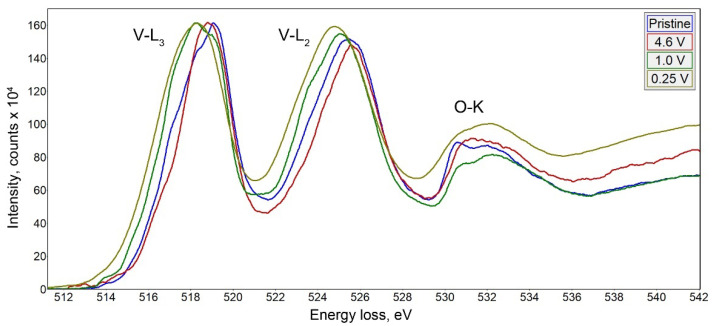
EELS spectra of Na_9_V_14_O_35_ at different states of charge in the vicinity of the V-L_3,2_ edge.

To draw the correlation between the structural transformation and change in the oxidation state of vanadium upon electrochemical cycling, we recorded EELS spectra in the vicinity of V-L_3,2_ edge (Figure 9). The spectra were interpreted in terms of correlation between the vanadium oxidation state and the onset of the V-L_3_ edge as proposed by Tan et al. [25]. The empirical dependence between the V-L_3_ edge onset E_V_ determined at 10% of the maximum height of the V-L_3_ edge, and the vanadium formal oxidation V_V_ state demonstrates a linear increase of E_V_ with V_V_. We have used the linear E_V_-V_V_ equation derived from a set of standard materials by Tan et al. [25] to estimate the vanadium oxidation state in the samples under investigation. In the pristine material, the onset energy is at E_V_ = 515.2 eV that corresponds to V_V_ = +4.3, in good agreement with the average oxidation state of +4.36 from the chemical composition Na_9_V^4.1+^_10_O_19_(V^5+^O_4_)_4_. Charging to 4.6 V vs. Na^+^/Na slightly shifts the V-L_3,2_ edge towards a higher energy loss resulting in E_V_ = 515.4 eV and V_V_ = +4.5 that corresponds to extraction of 3 Na per Na_9_V_14_O_35_ formula unit as deduced from the electrochemical data and EDX analysis (Na_6_V_14_O_35_, V_V_ = +4.57). Upon discharge to 1 V and 0.25 V, the V-L_3_ edge onset energy is reduced to E_V_ = 514.8 and 514.3 eV, respectively, providing V_V_ = +3.9 and +3.5. It should be noted that the vanadium reduction from +4.5 to +3.5 upon discharge from 4.6 V to 0.25 V accounts for only ~250 mAh g^−1^ capacity that corresponds to the end of the first discharge plateau. Thus, the capacity of the second and third discharge plateaus should be attributed to a conversion reaction with the formation of the Na-rich phase. Unfortunately, the two-phase nature of the sample discharged to 0.1 V and strong Na inhomogeneity even within the same crystallite (Figure S8) prevent sensible EELS measurement of V_V_.

## 4. Materials and Methods

### 4.1. Synthesis

Na_9_V_14_O_35_ contains vanadium in two oxidation states, 4+ and 5+. The synthesis in vacuum-sealed silica ampoules was implemented to stabilize vanadium in the intermediate oxidation state as it was previously reported by Millet at al. [22,23]. Initially, NaVO_3_ was synthesized by annealing the mixture of stoichiometric amounts of Na_2_CO_3_ (Ruskhim, 99%) and V_2_O_5_ (Sigma Aldrich, 99.6%) at 550 °C for 14 h. Single-phase polycrystalline Na_9_V_14_O_35_ was prepared from 3.06 mmol NaVO_3_, 0.05 mmol V_2_O_5_ and 0.8 mmol V_2_O_3_ (Alfa Aesar, 99.7%). The mixture of the initial reagents with the total weight of 0.5 g was ground thoroughly in an agate mortar, pressed into a 10 mm pellet, placed into an alumina crucible and sealed into a 16 mm quartz tube under dynamic vacuum of ~5∙10^−3^ mbar. The tube was heated with 120 °C/h heating rate to 650 °C, annealed for 50 h and cooled down with the furnace. The pellet was crushed, reground, pressed into a pellet and annealed at the same conditions for the second time.

### 4.2. Powder X-ray Diffraction

Powder X-ray diffraction (PXRD) pattern for the Rietveld analysis of the pristine Na_9_V_14_O_35_ crystal structure was collected with a STOE Stadi P diffractometer (STOE, Darmstadt, Germany) (transmission mode, Ge (111) monochromator, MYTHEN detector, Mo-Kα_1_ radiation, λ = 0.7093 Å). A Huber G670 Guinier camera equipped with a Ge (111) monochromator (Co-Kα_1_, λ = 1.7890 Å) and an image plate detector and a Bruker D8 ADVANCE (Cu-Kα, λ = 1.5425 Å) equipped with energy-dispersive LYNXEYE XE detector were also used for control of the phase purity. Electrode composites scratched from the Al current collector were studied at DESY synchrotron source (SXRD) at the P02.1 beamline (PETRA III, Hamburg, Germany) with λ = 0.20736(1) Å. Diffraction data recorded by the Perkin Elmer 2D detector (Perkin Elmer, Boston, MA, USA) were integrated and a wavelength was determined using reflection positions of LaB_6_ standard material. Rietveld refinement was performed using Jana2006 software [26].

### 4.3. Cells Assembling and Electrochemical Testing

Electrode composite was prepared from 80 wt.% Na_9_V_14_O_35_, 10 wt.% carbon black (Super P) as a conductive additive and 10 wt.% polyvinylidenefluoride (PVDF) as a binder. Prior to preparation of a slurry, active material mixed with carbon black and moistened with acetone was subjected to 1 h treatment in a high-energy SPEX-8000 ball mill (SPEX CertiPrep, Metuchen, NJ, USA) to reduce particle size and perform an effective mixing of all components. Then, PVDF was dissolved in N-methyl-2-pyrrolidone and mixed with the active material into slurry. The resulting slurry was coated on an aluminum foil with the Dr. Blade applicator and then dried in vacuum at 85 °C. Next, 16 mm circular electrodes made from the coated foil were dried in vacuum at 110 °C overnight.

The electrochemical cells were assembled in an argon-filled MBraun glove box (Mbraun, Garching, Germany)) with the residual oxygen and water content less than 0.1 ppm. A typical cell for galvanostatic cycling consisted of an electrode with the active material, a borosilicate glass fiber separator and sodium metal used as the counter electrode. Then, 1 M solution of NaClO_4_ in sulfolane:propylene carbonate mixed in 1:1 volume ratio was used as the electrolyte. Galvanostatic cycling with potential limitation was performed on a BioLogic potentiostat (BioLogic, Seyssinet-Pariset, France) at C/20, C/10 and C/5 rates, where 1C corresponds to the current density of 163 mA g^−1^ (removal/insertion of all sodium from/into the formula unit within one hour).

### 4.4. Scanning and Transmission Electron Microscopy

The morphology was characterized with a Quattro S ESEM scanning electron microscope (ThermoFisherScientific, Landsmeer, Netherlands). Samples for transmission electron microscopy were prepared by crushing the Na_9_V_14_O_35_ powder in an agate mortar in acetone followed by depositing the suspension onto holey TEM grid with a Lacey/Carbon-supporting layer. The electrodes at different state of charge were removed from electrochemical cells, double-washed in dimethyl carbonate (DMC) and scratched from the Al foil. All manipulations were performed in Ar-filled glove box followed by transportation to the TEM column by means of a dedicated vacuum holder, completely avoiding contact with air and moisture. Selected area electron diffraction (SAED) patterns, high-angle annular dark field scanning transmission electron microscopy (HAADF-STEM) and energy dispersive X-ray (EDX) compositional maps were taken with an aberration-corrected FEI Titan G3 transmission electron microscope (ThermoFisherScientific, Landsmeer, Netherlands) operated at 200 kV. The electron energy loss spectra (EELS) were registered in a STEM mode on a Titan Themis Z TEM (ThermoFisherScientific, Landsmeer, Netherlands) equipped with a Gatan Quantum ERS/966 P spectrometer (Gatan, München, Germany) and operated at 200 kV. Energy dispersion of 0.025 eV per channel was used; the energy resolution measured by full width at half maximum of zero-loss peak was 0.125 eV.

## 5. Conclusions

Na_9_V_14_O_35_ (η-Na_x_V_2_O_5_) has been synthesized by a solid-state route in an evacuated sealed silica tube and tested as electroactive material for Na half-cells. Being charged to 4.6 V vs. Na^+^/Na, almost 3 Na can be extracted per Na_9_V_14_O_35_ formula unit, resulting in a charge capacity of about 60 mAh g^−1^. Upon discharge below 1 V, Na_9_V_14_O_35_ uptakes Na up to the Na:V = 1:1 atomic ratio that is accompanied by a drastic increase of the separation between the layers of the VO_4_ tetrahedra and VO_5_ tetragonal pyramids, and a volume increase of about 31%. The induced structure instability triggers a transformation of the ordered layered Na_9_V_14_O_35_ structure into a rock-salt type disordered structure. Ultimately, the amorphous products of a conversion reaction are formed at 0.1 V, delivering the discharge capacity up to 490 mAh g^−1^, which, however, quickly fades with the number of charge-discharge cycles.

## Figures and Tables

**Table 1:** Crystallographic data and parameters of the Rietveld analysis of Na_9_V_14_O_35_.

Formula Unit	Na_9_V_14_O_35_
Space group	*P*2/*c*
*a*, Å	15.19901(17)
*b*, Å	5.03271(4)
*c*, Å	20.7739(2)
β, °	109.1635(6)
V, Å^3^	1501.0(4)
Z	2
ρ_calc_, g cm^−3^	3.2748
Parameters refined	100
Temperature, °C	25
Radiation	Mo-Kα
2θ range, step, deg.	5−70, 0.01
Number of reflections	4389
R_F_, R_P_, R_wP_	0.034; 0.055; 0.071

**Table 2:** Na:V atomic ratio calculated by STEM-EDX for Na_9_V_14_O_35_ at different states of (dis)charge.

Charge/Discharge Voltage	Na:V Ratio ^1^
pristine	8.8(2):14.0
4.6 V	6.0(4):14.0
0.25 V	15.0(9):14.0
0.1 V	Phase 1: 13.9(3):14.0Phase 2: 94(3):14.0

^1^ All data are normalized to 14 atoms of V (i.e., per one Na_9_V_14_O_35_ formula unit).

## Data Availability

Relevant data are contained within the article and the Supporting Information.

## Supplementary Materials

The following are available online, Figure S1: Experimental and calculated PXRD profiles (and their difference) after Rietveld refinement of Na_9_V_14_O_35_. The black ticks indicate the Bragg reflection positions, Figure S2: HAADF-STEM image, individual Na, V and mixed Na/V EDX maps of the pristine Na_9_V_14_O_35_ material; Figure S3: Initial charge-discharge curves of the ball-milled Na_9_V_14_O_35_ sample cycled with a rate of 16.3 mA g^−1^ (corresponding to C/10 as it is defined in the main text) (potential window 1.5–4.8 V vs. Na^+^/Na); Figure S4: Galvanostatic cycling for Na_9_V_14_O_35_ in Na cell in a potential window of 0.1–3.0 V vs. Na^+^/Na (a) and comparison of specific capacity in the different potential windows at C/10 rate (b); Figure S5: Experimental and calculated profiles (and their difference) after a Le Bail analysis of the SXRD pattern of Na_9_V_14_O_35_ discharged to 1 V vs. Na^+^/Na. The black ticks indicate the Bragg reflection positions of the *P*2/*c* unit cell. Wavelength λ = 0.20736 Å; Figure S6: [010] SAED patterns of pristine (a) and discharged to 1 V (c) Na_9_V_14_O_35_. High-resolution [010] HAADF-STEM images of pristine (b) and discharged to 1 V (d) Na_9_V_14_O_35_; Figure S7: Experimental and calculated profiles (and their difference) after Le Bail analysis of the SXRD pattern of Na_9_V_14_O_35_ discharged to 0.25 V vs. Na^+^/Na. The black ticks indicate the Bragg reflection positions of the *P*2/*c* unit cell. Wavelength λ = 0.20736 Å; Figure S8: HAADF-STEM images, individual Na, V and mixed Na/V EDX maps of Na_9_V_14_O_35_ discharged to 0.1 V, Table S1: Fractional atomic coordinates and occupancies for pristine Na_9_V_14_O_35_, Table S2: Selected interatomic distances for pristine Na_9_V_14_O_35_ (Å).
