# Supplementary material for: Sodium-Vanadium Bronze Na9V14O35: An Electrode Material for Na-Ion Batteries
Source: Molecules. 2021 Dec 24;27(1):86. doi: 10.3390/molecules27010086 (PMC8747075; doi:10.3390/molecules27010086)
Supplement: Supplementary file 1 [file molecules-27-00086-s001.zip › molecules-1523675-supplementary.pdf]

Supplementary information for the manuscript "Sodium-vanadium bronze $Na_9V_{14}O_{35}$: an electrode material for Na-ion batteries"

Maria A. Kirsanova, Alexey S. Akmaev, Mikhail V. Gorbunov, Daria Mikhailova and Artem M. Abakumov*

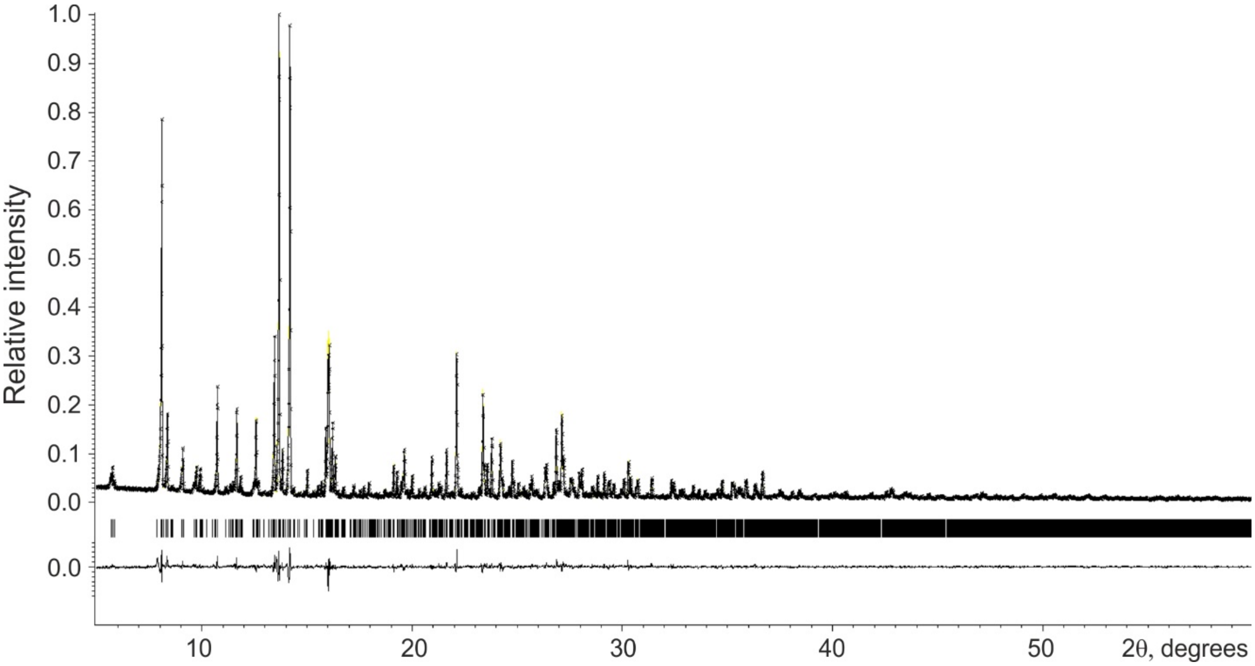

Figure S1. Experimental and calculated PXRD profiles (and their difference) after Rietveld refinement of $Na_9V_{14}O_{35}$. The black ticks indicate the Bragg reflection positions.

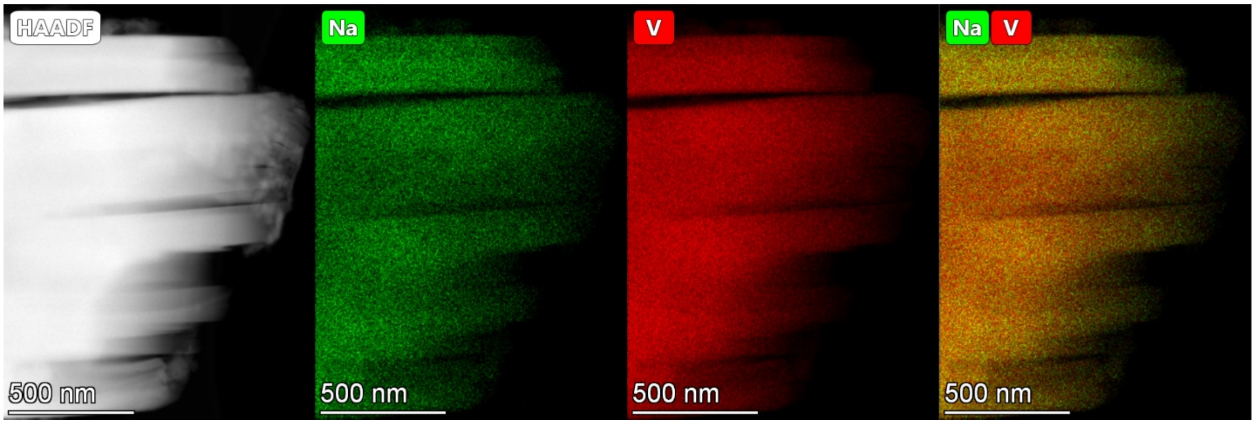

Figure S2. HAADF-STEM image, individual Na, V and mixed Na/V EDX maps of the pristine $Na_9V_{14}O_{35}$ material.

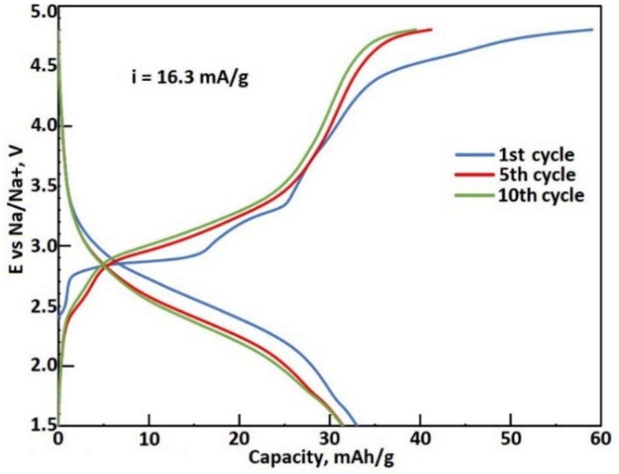

Figure S3. Initial charge-discharge curves of the ball-milled $Na_9V_{14}O_{35}$ cycled with a rate of 16.3 mA g^{-1} (corresponding to C/10 as it is defined in the main text) (potential window 1.5-4.8 V vs Na^+/Na).

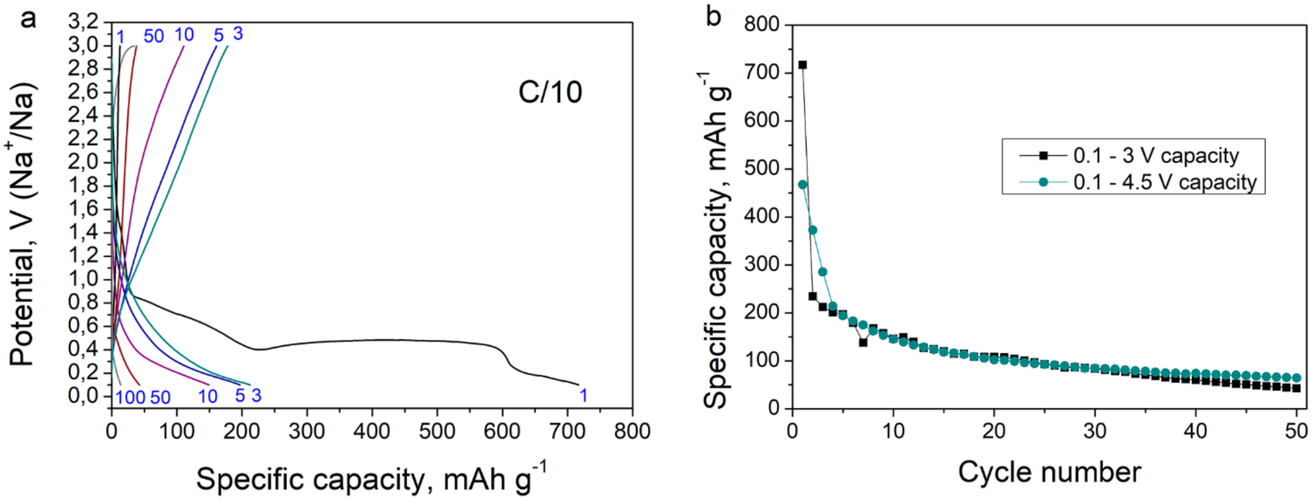

Figure S4. Galvanostatic cycling for $Na_9V_{14}O_{35}$ in Na cell in a potential window of 0.1–3.0 V vs Na^+/Na (a) and comparison of specific capacity in the different potential windows at C/10 rate (b).

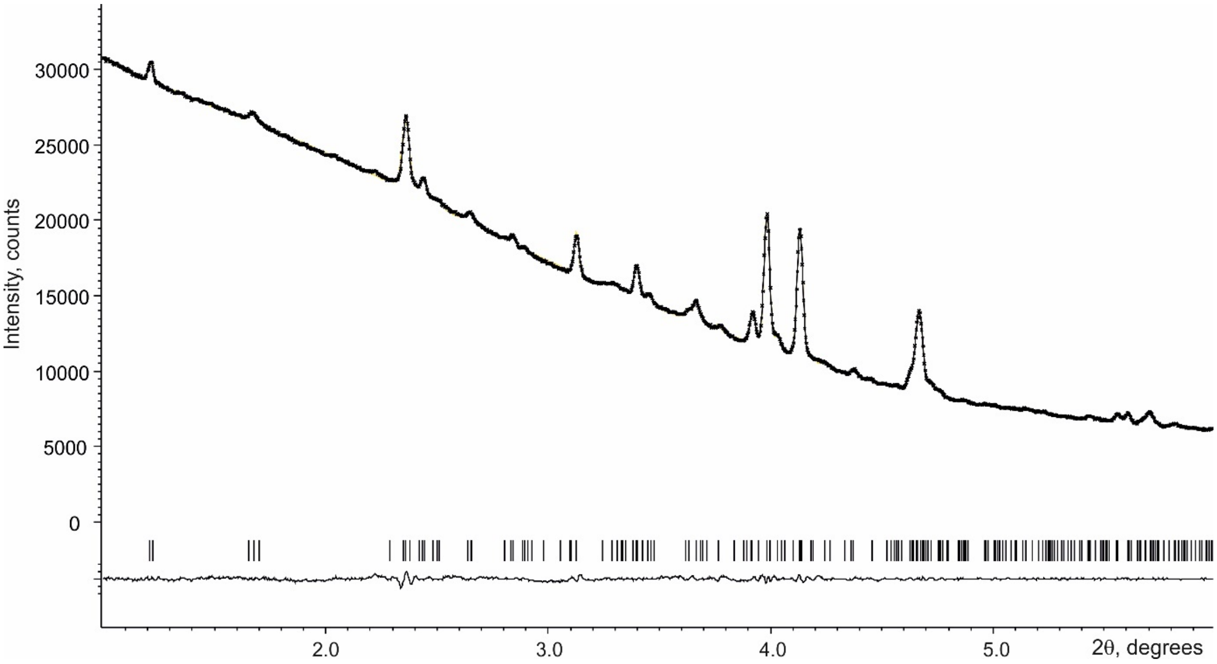

Figure S5. Experimental and calculated profiles (and their difference) after a Le Bail analysis of the SXRD pattern of $Na_9V_{14}O_{35}$ discharged to 1 V vs Na^+/Na. The black ticks indicate the Bragg reflection positions of the *P*2/*c* unit cell. Wavelength λ = 0.20736 Å.

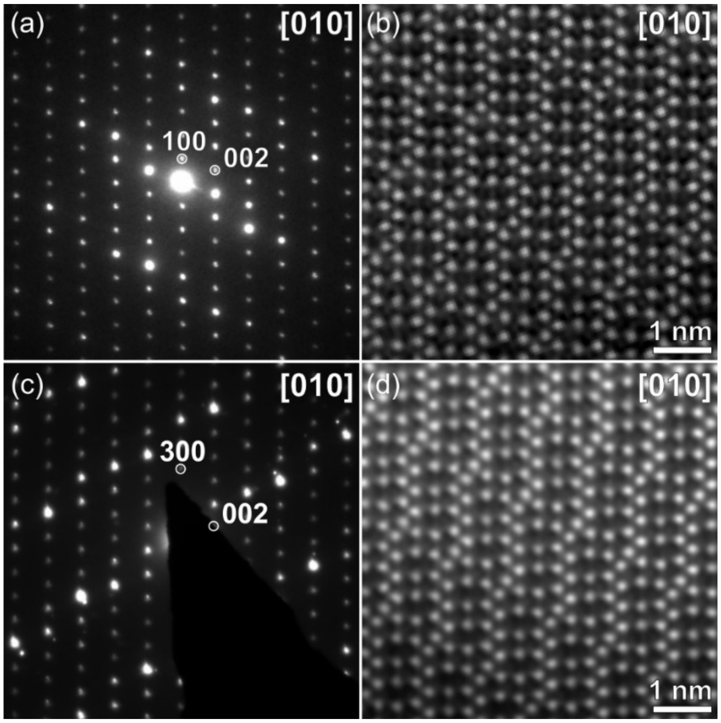

Figure S6. [010] SAED patterns of pristine (a) and discharged to 1 V (c) $Na_9V_{14}O_{35}$. High-resolution [010] HAADF-STEM images of pristine (b) and discharged to 1 V (d) $Na_9V_{14}O_{35}$.

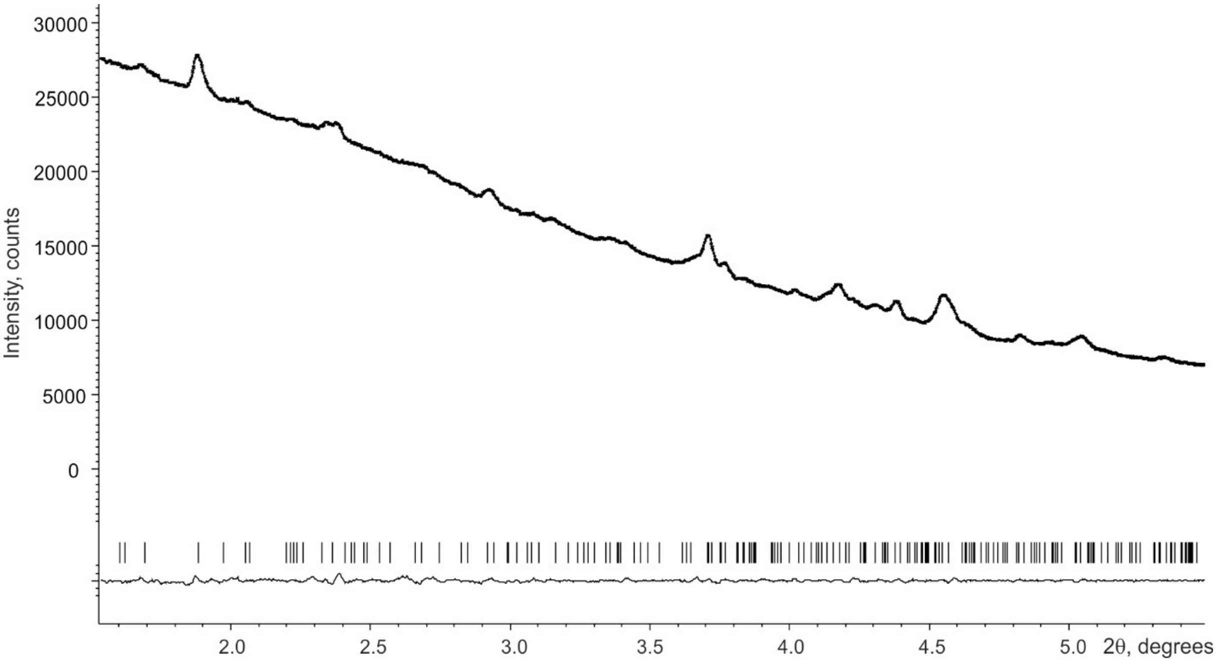

Figure S7. Experimental and calculated profiles (and their difference) after Le Bail analysis of the SXRD pattern of $Na_9V_{14}O_{35}$ discharged to 0.25 V vs Na^+/Na. The black ticks indicate the Bragg reflection positions of the *P*2/*c* unit cell. Wavelength λ = 0.20736 Å.

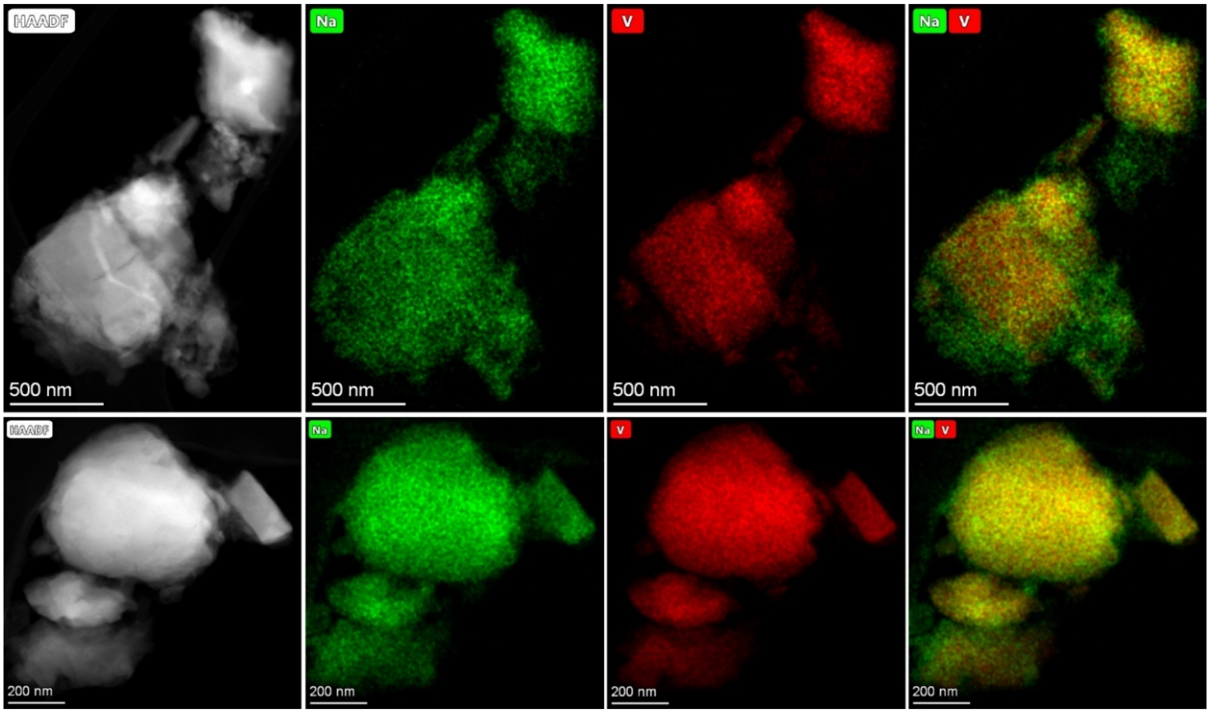

Figure S8. HAADF-STEM images, individual Na, V and mixed Na/V EDX maps of $Na_9V_{14}O_{35}$ discharged to 0.1 V.

Table S1. Fractional atomic coordinates and occupancies for pristine $Na_9V_{14}O_{35}$.

Atom	Position	*x/a*	*y/b*	*z/c*	U_{iso}, Å^2
V1	4*g*	0.5132(3)	0.3955(12)	0.4358(2)	0.0042(14)
V2	4*g*	0.7250(3)	0.3999(12)	0.3940(2)	0.0113(14)
V3	4*g*	0.9351(3)	0.4162(10)	0.3493(2)	0.0016(12)
V4	4*g*	0.1465(3)	0.4166(10)	0.3183(2)	0.0075(12)
V5	4*g*	0.9068(3)	0.6180(10)	0.4777(2)	0.0075(13)
V6	4*g*	0.3013(3)	0.4038(10)	0.4779(2)	0.0036(12)
V7	4*g*	0.4027(3)	0.3522(7)	0.2676(2)	0.0049(10)
Na1	4*g*	0.4362(7)	0.129(2)	0.6517(5)	0.012(3)
Na2	4*g*	0.2151(7)	0.117(2)	0.6991(5)	0.006(2)
Na3	4*g*	0.6547(8)	0.115(2)	0.6220(5)	0.016(3)
Na4	4*g*	0.8707(7)	0.136(2)	0.5790(5)	0.014(3)
Na5	2*e*	0	0.097(3)	0.75	0.026(5)
O1	4*g*	0.5875(10)	0.460(3)	0.5345(8)	0.0072(7)
O2	4*g*	0.5007(10)	0.079(4)	0.4184(8)	0.0072(7)
O3	4*g*	0.3746(12)	0.462(3)	0.5748(8)	0.0072(7)
O4	4*g*	0.7990(11)	0.476(3)	0.4867(8)	0.0072(7)
O5	4*g*	0.2948(10)	0.070(4)	0.4673(7)	0.0072(7)
O6	4*g*	0.7156(10)	0.090(4)	0.3794(7)	0.0072(7)
O7	4*g*	0.1580(11)	0.464(3)	0.6112(7)	0.0072(7)
O8	4*g*	0.5504(11)	0.454(3)	0.6543(8)	0.0072(7)
O9	2*f*	0.5	0.190(4)	0.25	0.0072(7)
O10	4*g*	0.1413(10)	0.121(4)	0.3226(6)	0.0072(7)
O11	4*g*	0.9294(9)	0.087(4)	0.3387(7)	0.0072(7)
O12	4*g*	0.1438(9)	0.475(3)	0.7427(7)	0.0072(7)
O13	4*g*	0.3492(11)	0.459(3)	0.6967(8)	0.0072(7)
O14	4*g*	0.7700(11)	0.449(3)	0.6141(8)	0.0072(7)
O15	4*g*	0.3351(9)	0.103(3)	0.2829(7)	0.0072(7)
O16	4*g*	0.9581(10)	0.458(3)	0.6743(6)	0.0072(7)
O17	4*g*	0.0932(9)	0.079(3)	0.5076(7)	0.0072(7)
O18	4*g*	0.9948(10)	0.468(3)	0.5546(7)	0.0072(7)

Table S2. Selected interatomic distances for pristine $Na_9V_{14}O_{35}$ (Å).

V1-O1	1.969(18)	V6-O1	1.918(18)
V1-O2	1.629(18)	V6-O3	1.975(15)
V1-O3	1.93(2)	V6-O4	1.993(19)
V1-O8	1.955(16)	V6-O5	1.689(18)
V2-O3	1.96(2)	V6-O14	2.003(15)
V2-O4	1.922(15)	V7-O8	1.826(16)
V2-O6	1.58(2)	V7-O9	1.830(10)
V2-O7	1.942(18)	V7-O13	1.716(16)
V2-O13	1.982(16)	V7-O15	1.715(16)
V3-O7	1.952(18)	Na1-O2	2.25(2)
V3-O11	1.669(18)	Na1-O3	2.294(19)
V3-O12	1.971(13)	Na1-O8	2.37(2)
V3-O16	1.949(17)	Na2-O6	2.45(2)
V3-O18	2.016(13)	Na2-O11	2.315(17)
V4-O10	1.492(18)	Na2-O12	2.423(19)
V4-O12	1.651(16)	Na2-O15	2.343(15)
V4-O14	1.692(14)	Na3-O2	2.433(19)
V4-O16	1.763(16)	Na3-O5	2.42(2)
V5-O4	1.853(18)	Na3-O15	2.220(19)
V5-O7	1.833(14)	Na4-O17	2.31(2)
V5-O17	1.553(18)	Na5-O10	2.444(16)
V5-O18	1.870(13)	Na5-O16	2.350(19)
